# Correction of CFTR function in nasal epithelial cells from cystic fibrosis patients predicts improvement of respiratory function by CFTR modulators

**DOI:** 10.1038/s41598-017-07504-1

**Published:** 2017-08-07

**Authors:** Iwona M. Pranke, Aurélie Hatton, Juliette Simonin, Jean Philippe Jais, Françoise Le Pimpec-Barthes, Ania Carsin, Pierre Bonnette, Michael Fayon, Nathalie Stremler-Le Bel, Dominique Grenet, Matthieu Thumerel, Julie Mazenq, Valerie Urbach, Myriam Mesbahi, Emanuelle Girodon-Boulandet, Alexandre Hinzpeter, Aleksander Edelman, Isabelle Sermet-Gaudelus

**Affiliations:** 1 Inserm U1151 - CNRS UMR 8253 – team 2, Faculté de Médecine Paris Descartes, Paris, France; 2 Biostatistics Department, Hôpital Necker Enfants Malades, Assistance Publique Hôpitaux de Paris, Paris, France; 3 Service de Chirurgie Thoracique, Hôpital Européen Georges Pompidou, Assistance Publique Hôpitaux de Paris, Paris, France; 4 Service de Pneumo-Pédiatrie, Hôpital de la Timonne, Marseille, France; 5 Service de Pneumologie, Hôpital Foch, Suresnes, France; 6 Service de Pneumo-Pédiatrie, Hôpital Pellegrin, Bordeaux, France; 7 Service de Chirurgie Thoracique, Hôpital Pellegrin, Bordeaux, France; 8 Service de génétique et biologie moléculaires, Hôpital Cochin, Assistance Publique Hôpitaux de Paris, Paris, France; 9 Cystic Fibrosis Center, Hôpital Necker Enfants Malades, Assistance Publique Hôpitaux de Paris, Paris, France

## Abstract

Clinical studies with modulators of the Cystic Fibrosis Transmembrane conductance Regulator (CFTR) protein have demonstrated that functional restoration of the mutated CFTR can lead to substantial clinical benefit. However, studies have shown highly variable patient responses. The objective of this study was to determine a biomarker predictive of the clinical response. CFTR function was assessed *in vivo* via nasal potential difference (NPD) and in human nasal epithelial (HNE) cultures by the response to Forskolin/IBMX and the CFTR potentiator VX-770 in short-circuit-current (∆I_scF/I+V_) experiments. CFTR expression was evaluated by apical membrane fluorescence semi-quantification. I_sc_ measurements discriminated CFTR function between controls, healthy heterozygotes, patients homozygous for the severe F508del mutation and patients with genotypes leading to absent or residual function. ∆I_scF/I+V_ correlated with CFTR cellular apical expression and NPD measurements. The CFTR correctors lumacaftor and tezacaftor significantly increased the ∆I_scF/I+V_ response to about 25% (SEM = 4.4) of the WT-CFTR level and the CFTR apical expression to about 22% (SEM = 4.6) of the WT-CFTR level in F508del/F508del HNE cells. The level of CFTR correction in HNE cultures significantly correlated with the FEV_1_ change at 6 months in 8 patients treated with CFTR modulators. We provide the first evidence that correction of CFTR function in HNE cell cultures can predict respiratory improvement by CFTR modulators.

## Introduction

Cystic Fibrosis (CF) is the most frequent lethal autosomic disease in the Caucasian population. In its typical form it induces the production of a highly salted sweat, pancreatic insufficiency and a progressive destructive infected bronchopathy which leads to respiratory failure^1^. CF is caused by mutations in the Cystic Fibrosis Transmembrane Conductance Regulator (*CFTR*) gene coding for the CFTR protein, a member of the ATP-binding cassette transporter family of membrane proteins. It forms a small-conductance, chloride (Cl^−^) channel that is gated by protein kinase A-mediated phosphorylation, and ATP binding/ hydrolysis^2^.

The most frequent mutation, the deletion of one amino-acid phenylalanine at position 508, is associated with misfolding of the protein leading to its degradation by the proteasomal pathway. Corrector therapy aims to stabilize the folding of the protein and limit its degradation. As a result, little to no F508del-CFTR is delivered to the cell surface, and CFTR Cl^−^ transport activity is abolished^2^. Other mutations limit the gating of the channel, such as the G551D mutation, and their function is restored by therapies potentiating the opening of the channel.

A recent proof-of-concept study on the CFTR potentiator ivacaftor (VX-770)^3^ has demonstrated that pharmacological manipulation of the G551D-CFTR protein can translate into clinical benefit for CF patients and potentially profoundly modify the disease prognosis^4, 5^. This has also been demonstrated for other malfunctioning proteins affecting the gating of the channel^6^. Improvement of the *in vitro* activity of F508del-CFTR requires the combination of lumacaftor (VX-809)-CFTR corrector^7^ and ivacaftor CFTR potentiator^3^. However, such therapy is associated with variable clinical responsiveness. This was illustrated in a therapeutic trial testing the lumacaftor and ivacaftor combination (Orkambi) in F508del homozygous patients^8^. Indeed, only 25% of the subjects showed respiratory function improvement greater than 10% after 6 months of treatment, suggesting that the efficacy of CFTR modulators may differ according to the patient^8^. Different hypothesis have been proposed to explain the variable efficacy of Orkambi therapy, including destabilization of lumacaftor rescued F508del-CFTR by chronic ivacaftor treatment^9, 10^, the rescue of a subpopulation of F508del-CFTR Cl^−^ channels^11^, and action of Orkambi on other factors than transepithelial ion transport such as mucociliary clearance^12^. These observations highlight the importance of finding biomarkers that can predict the individual patients’ responses.

As the main goal of CFTR modulator therapy is to retard the disease progression in patients’ lungs, scientists have focused on respiratory epithelial cells. Human bronchial epithelial (HBE) cells have been used to assess the efficacy of CFTR modulators *in vitro* ^3, 7, 9, 10, 13–16^. However, these cells cannot be used routinely, as they are derived from explanted lungs. To overcome this problem, human nasal epithelial (HNE) cell cultures have been developed. These are easy to collect by simple nasal brushing and allow quantification of cyclic AMP (cAMP)-mediated Cl^−^ transport as a surrogate marker of CFTR function.

Although pioneering studies have shown that primary HNE cells recapitulate properties of lower airway epithelial cells^17–19^, there are no data showing their ability to distinguish levels of CFTR function according to CF genotype. Moreover, the correlation between CFTR bioactivity assessed *in vitro* in this model and *in vivo* in the nasal mucosa of the same patients has never been investigated. Furthermore, no systematic study has assessed the variability of HNE cell responses to CFTR correctors. Finally, and most importantly, whether the rescue of CFTR activity with CFTR modulators in this model is predictive of the clinical efficacy in patients, needs to be determined.

In this study, we first characterized CFTR expression and function in HNE cells isolated from CF patients, healthy controls, and heterozygotes to correlate *in vitro* functional data with *in vivo* nasal potential difference measurement elicited by cAMP agonists. We then undertook a systematic study to evaluate the correction of CFTR function by two CFTR correctors, lumacaftor and tezacaftor (VX-661), in HNE cultures issued from patients homozygous for the F508del mutation or carrying *CFTR* genotypes displaying a wide spectrum of CFTR activity. Finally, we assessed the predictive value of the primary HNE cell cultures by comparing the pharmacological rescue of CF mutations by CFTR modulators *in vitro* with the clinical efficacy of these agents in CF patients.

## Results

### HNE cell cultures recapitulate the properties of HBE cell cultures

We characterized the properties of non-expanded cultures at passage 0 to remain as close as possible to the *in vivo* physiology. Both primary HNE and HBE cells displayed typical features of polarized and differentiated respiratory epithelium (Supplementary Fig. S1a,b). The ciliated cells were the predominant type of cells, accounting for 47–91% of all types of cells in HNE cultures, as assessed by the percentage of cells with alpha-tubulin staining (Supplementary Fig. S1a,b).

Short-circuit-current (I_sc_) experiments showed statistically significant differences between wt/wt and F508del/F508del HNE cultures for ∆I_sc_ changes induced by Forskolin/IBMX+VX-770 and CFTR_inh_172, similarly to HBE cell cultures (Fig. 1a,b; Supplementary Fig. S1c; Table 1). The only difference between HNE and HBE cell cultures, although not statistically significant, was the mean basal current, which was associated with an increased response to Amiloride (∆I_scAmiloride_) in wt/wt HBE *versus* HNE cells (Fig. 1a,b; Table 1). The wt/F508del HNE cells presented basal currents (19.4 ± 6.2 µA/cm^2^) and ∆I_scAmiloride_ (−8.8 ± 4.7 µA/cm^2^) that were not statistically different from the responses observed in wt/wt cells. In contrast, the responses to Forskolin/IBMX + VX-770 (∆I_scF/I+V_ 5.1 ± 1.4 µA/cm^2^) and CFTR_inh_172 (∆I_scCFTRinh172_ −9.2 ± 2.4 µA/cm^2^) were significantly different from both the wt/wt and F508del homozygous HNE cells (Fig. 1b). The variation of ∆I_sc_ after Forskolin/IBMX + VX-770, which is the main endpoint for corrector efficacy assessment, was analyzed to gain further insight into inter- and intra-subject variability. Both in wt/wt and F508del homozygous cell cultures, the inter-subject variability accounted for around 2/3 of the total variability with 67% of total variance for wt/wt cells (inter-subject variability 0.47 and intra-subject variability 0.23; Intra-Class Correlation ICC = 0.47/(0.47 + 0.23) = 0.67), and 70% for F508del/F508del cells (inter-subject variability 0.6 and intra-subject variability 0.23; ICC = 0.6/(0.6 + 0.23) = 0.7), as estimated with linear random effect model, with no significant difference between HNE and HBE cells.

**Figure 1:**
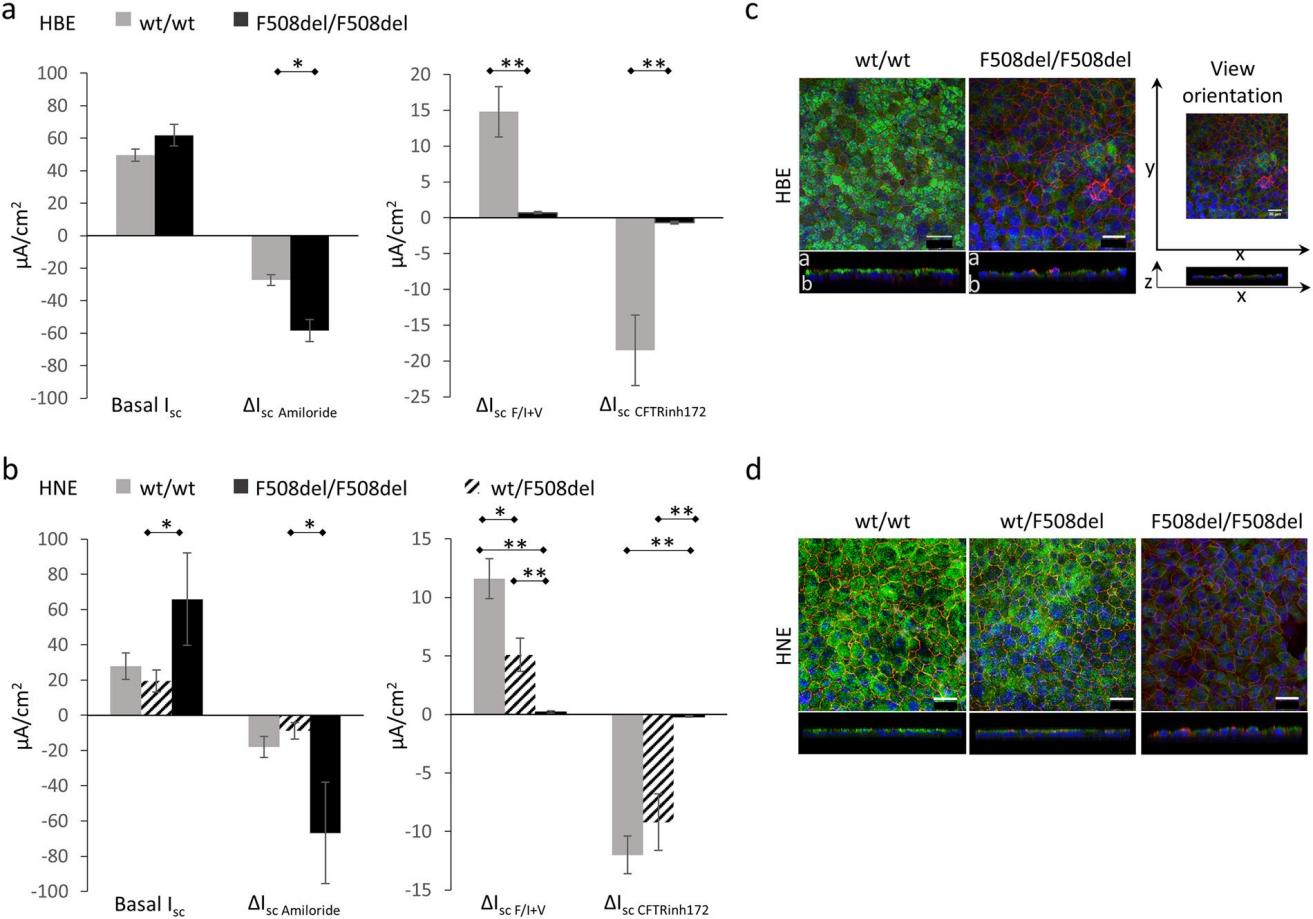
Characteristics of the trans-epithelial ion transport and CFTR expression in HBE and HNE cell cultures. Mean and SEM values for Basal I_sc_, ∆I_scAmiloride_, ∆I_scF/I+V_ and ∆I_scCFTRinh172_ (µA/cm^2^) were calculated from median values per patient. Comparison by non-parametric Mann Whitney test. At least 2 filters were analyzed per patient. The *p*-values for statistically significant differences are indicated as * < 0.05 and ** < 0.01. (**a**) Comparison of wt/wt (grey) and F508del/F508del (black) HBE cell cultures issued from non-expanded cells. (**b**) Comparison of wt/wt (grey), wt/F508del (dashed), and F508del/F508del (black) HNE cell cultures issued from non-expanded cells. Representative confocal microscopy images of immuno-fluorescent staining of CFTR (green) and Zona-Occludens-1 (red) in HBE (**c**) and HNE (**d**) cell cultures according to genotypes. Top and side views of three-dimensional (3D) reconstitutions (a: apical face, b: basal face of epithelium). Scale bars = 20 µm.

**Table 1:** Comparison of HBE and HNE cell cultures issued from healthy donors (wt/wt) and F508del homozygous patients (F508del/F508del). Mean (±SEM) were calculated from median values per patient. Comparison by non-parametric Mann-Whitney test. ∆I_sc_ results are expressed in µA/cm^2^.

	HBE			HNE			HBE - HNE	
	wt/wt	F508del/F508del	p*	wt/wt	F508del/F508del	p*	p**	P***
	N = 5	N = 11		N = 7	N = 5			
	n = 16	n = 24		n = 17	n = 12			
Basal I_sc_	49.6 (3.8)	61.8 (6.6)	NS	27.9 (7.6)	65.9 (26.3)	NS	NS	NS
∆I_sc Amiloride_	−27.3 (3.4)	−58.35 (6.7)	0.01	−17.9 (6)	−66.8 (28.8)	NS	NS	NS
∆I_sc F/I+V_	14.8 (3.5)	0.7 (0.16)	<0.01	11.6 (1.7)	0.2 (0.09)	0.01	NS	NS
∆I_sc CFTRinh172_	−18.5 (4.9)	−0.77 (0.2)	<0.01	−12 (1.6)	−0.17 (0.05)	0.01	NS	NS
Percentage of cells with apical staining of CFTR	86.5% (2.3)	9.9% (3.1)	0.02	85.5% (0.5)	7.3% (1.2)	0.04	NS	NS
Average corrected apical fluorescence of CFTR staining	37 × 10^5^ (2.3 × 10^5^)	6.8 × 10^5^ (3 × 10^5^)	0.02	30 × 10^5^ (0.3 × 10^5^)	8 × 10^5^ (2.2 × 10^5^)	0.04	NS	NS

Data are presented from n filters obtained from N subjects. At least 2 filters per patient were analyzed. p*: wt/wt – F508del/F508del; p**: wt/wt HNE – wt/wt HBE; p***: F508del +/+ HNE – F508del +/+ HBE.

There was no significant difference for the CFTR staining patterns between HNE and HBE cells (Fig. 1c,d). Apical CFTR staining was observed in 86% (83–91%) of wt/wt HNE and HBE cells, whereas it was present in only 8% (3–18%) of F508del cells. The average corrected apical fluorescence was around 5-fold weaker in F508del homozygous cells than in wt/wt cells (Table 1). The CFTR staining was intermediate in wt/CF HNE cells with 57% (49–67%) of positive cells, but the average corrected apical fluorescence was close to normal (Fig. 1d). The proportion of cells with apical immunostaining was significantly correlated with the average corrected apical fluorescence (Supplementary Fig. S2 **)**.

### Correlation between CFTR-dependent Cl^−^ secretion in primary HNE cell cultures and nasal epithelia *in vivo*

To gain further insight into the characterization of the HNE model, we compared the ∆I_scF/I+V_ changes in cell cultures from patients with genotypes associated with a wide range of functional defects to the mean ∆I_scF/I+V_ value in wt/wt cells.

As expected, the genotypes including premature termination codon (PTC) (Y122X, G542X), deletion generating PTC (394delTT), severe splicing mutations (711 + 1 G > T, 1717 1 G > A), and the misfolding N1303K mutation, did not display CFTR-dependent Cl^−^ transport, whereas the terminally truncated mutation (E1418X), the mild splicing mutation (2789 + 5 G > A), and mutations associated with atypical CF such as D1152H, displayed residual function (Fig. 2, x-axis). Finally, two genotypes involving mutations of uncertain liability L997F/R258G^20^ and G1244E/R352Q^21^ (Supplementary Fig. S7) displayed WT level activity. This gradient in residual Cl^−^ transport was significantly correlated to apical CFTR staining (Fig. 2, y-axis; Supplementary Fig. S2).

**Figure 2:**
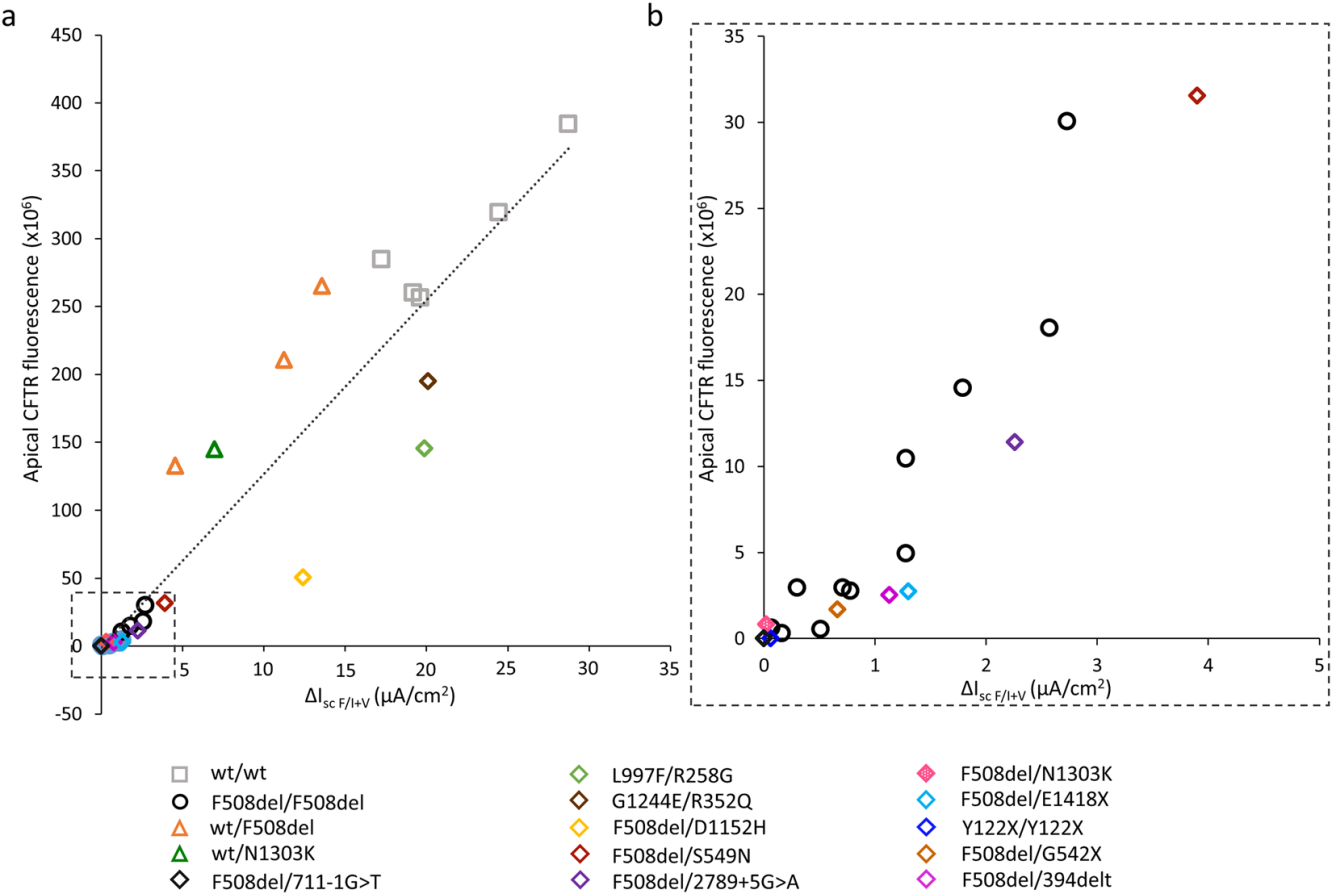
Correlation between cAMP dependent chloride transport and apical CFTR expression in HNE cells according to genotype. cAMP-dependent chloride transport is expressed as the I_sc_ response (∆I_sc_) to Forskolin (10 µM)/IBMX (100 µM) + VX-770 (10 µM) (∆I_scF/I+V_). The apical CFTR staining is expressed as the apical CFTR fluorescence (average corrected apical fluorescence multiplied by the percentage of cells with apical staining). The intensity of fluorescence was evaluated in the apical zone of epithelial cells and the region outside the cells for background correction in at least 3 cells from 3 random fields. Data from 5 wt/wt control subjects, 11 F508del/F508del patients, 3 wt/F508del ( + 1 wt/N1303K) heterozygotes, and 11 patients with other genotypes (legend in the figure). The dotted line shows simple regression analysis of the data (*R* ^*2*^ = 0.9, *p* < 0.0001). (**a**) Correlation graph representing all data. (**b**) Inset for patients with ∆I_scF/I+V_ below 5 µA/cm^2^. Inset shown in graph (**a**) by dashed line square.

To elucidate the physiological relevance of the HNE cell culture model, we compared ∆I_scF/I+V_ to the response to low-Cl^−^ solution and Isoproterenol, as assessed by nasal potential difference (NPD) in the same patient (Fig. 3, Supplementary Figs S5, S6 and S7). The correlation was high (*R* ^*2*^ = 0.82, *p* < 0.0001), indicating that the primary HNE reproduce the *in vivo* detected differences in the mutant CFTR activity.

**Figure 3:**
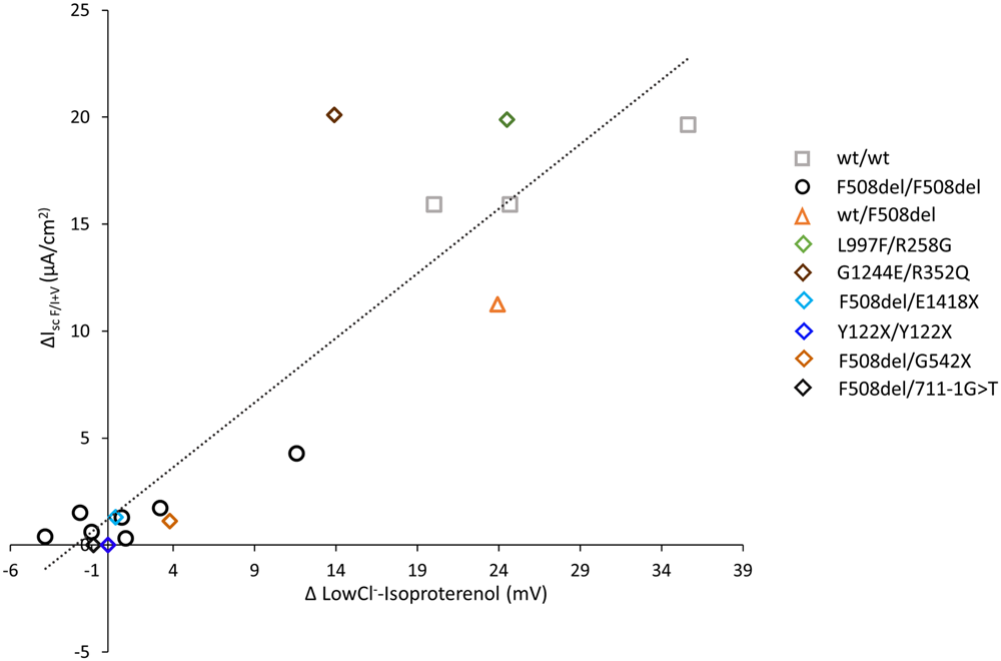
Correlation between CFTR activity evaluation in nasal cells *in vivo* and *in vitro* according to genotype. CFTR activity *in vivo* was expressed as the modification of the transepithelial potential difference in response to the sequential perfusion of the nasal mucosa to low Cl^−^ solution and the further addition of Isoproterenol 10 µM (∆LowCl^−^Isoproterenol). CFTR activity *in vitro* was expressed as the I_sc_ response to Forskolin (10 µM)/IBMX (100 µM) + VX-770 (10 µM) (∆I_scF/I+V_). Data from 3 wt/wt control subjects, 1 wt/F508del heterozygote, 7 F508del/F508del, and 6 patients with other genotypes (legend in the figure). The dotted line shows simple regression analysis of the data (*R* ^*2*^ = 0.82, *p* < 0.0001).

In contrast to Cl^−^, the Na^+^ transport, evaluated by the response to Amiloride during NPD and I_sc_, did not display such variation (*R* ^*2*^ = 0.04, NS; simple regression analysis), and it was not correlated to ∆I_scF/I+V_ values (*R* ^*2*^ = 0.0004, NS; simple regression analysis).

### Lumacaftor and tezacaftor correction of F508del CFTR is variable in conditionally reprogrammed HNE cultures from F508del homozygous patients

The effect of lumacaftor was evaluated in conditionally reprogrammed HNE (CR-HNE) cultures from 11 F508del homozygous patients and in HBE cultures from 11 F508del homozygous patients. To increase the number of filters available, we used conditionally reprogrammed and re-differentiated HNE cells but remained at passage 1 to maintain comparable CFTR expression to that observed in non-amplified cells. Both in patients homozygous for F508del and wt/wt subjects, the magnitude of I_sc_ changes to Amiloride, Forskolin/IBMX+VX-770 and CFTR_inh_172 in CR-HNE were not significantly different from those obtained in primary non-expanded HNE cells (e.g. ∆I_scF/I+V_ = 0.8 µA/cm^2^ (range min-max 1.46) in non-treated F508del/F508del CR-HNE n = 15; and 0.2 µA/cm^2^ (range min-max 0.58) in non-treated non-reprogrammed HNE n = 12).

Lumacaftor treatment significantly increased the average ∆I_scF/I+V_ in HNE and HBE cells as compared to DMSO by a mean of 1.8 µA/cm^2^ (SEM = 0.5). This was evaluated in 22 different patients (in 44 DMSO treated and 44 lumacaftor treated inserts). This correction allowed to reach an average of 25% (SEM = 4.4) of the WT-CFTR level (*p* < 10^−6^) (Fig. 4a). Lumacaftor was able to correct CFTR activity above 10% of the WT-CFTR, a level observed in patients with mild disease^22–24^, for 16 patients out of the 22 tested, and above the mean level obtained in healthy heterozygotes for 4 patients.

**Figure 4:**
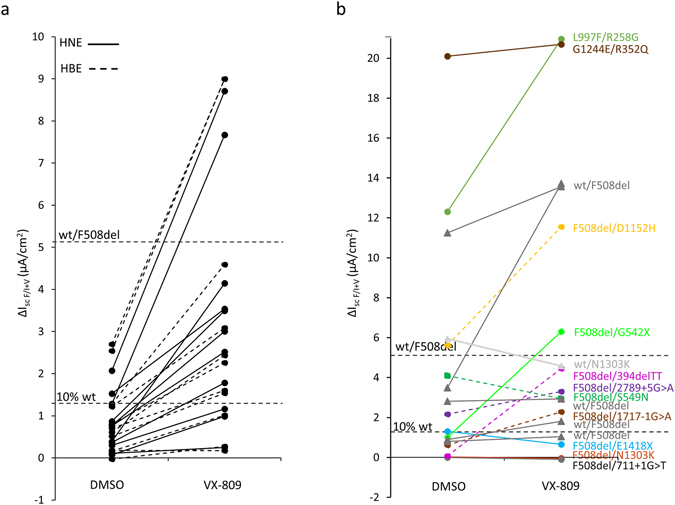
Lumacaftor stimulated correction of CFTR-dependent Cl^−^ secretion. I_sc_ response to Forskolin (10 µM)/IBMX (100 µM) + VX-770 (10 µM) (∆I_scF/I+V_) after treatment for 48 h at 37 °C with DMSO or lumacaftor 3 µM of HNE (solid line) and HBE (dashed line) cell cultures from different F508del/F508del patients (**a**) and patients with other genotypes (**b**). Specific genotypes are indicated and color coded. Each pair of points corresponds to an individual patient. Supplemental horizontal dashed lines in the graphs indicate the level of mean ∆I_scF/I+V_ in wt/F508del cultures sampled from healthy carriers and 10% of the mean ∆I_scF/I+V_ value in wt/wt cultures corresponding to the level of CFTR activity observed in patients with mild CF disease.

The variability of the treatment effect was mainly due to inter-patient variability (0.87) rather than intra-patient variability (0.25), which accounted for 77% of the total variability (intra-class correlation coefficient ratio of inter-patient variability over inter + intra-patient variability), as estimated with linear random effect model. However, the lumacaftor-induced change was not significantly different in HBE *versus* HNE cultures. The inter-patient variability was not associated with increased local nasal inflammation, as assessed by local visualization before nasal cell sampling, nor patients’ sputum colonization. The quantification of the VX-809 correction variability enabled us to calculate the number of filters necessary to assess the effect of CFTR corrector in F508del/F508del patients with a reliable power (Supplementary Table 1).

Tezacaftor was evaluated in HNE and HBE cultures of 14 F508del homozygous patients. It induced a significant increase of ∆I_scF/I+V_ by a mean of 2.5 µA/cm^2^ up to 27.4% of the WT-CFTR (SEM = 3.8; *p* = 0.001) (Supplementary Fig. S3a). A correction of the Cl^−^ secretion above 10% of the WT average, was obtained in 13 out of 14 patients, and over the healthy heterozygote range in 2 patients.

### Lumacaftor and tezacaftor induced CFTR correction in other genotypes

The Forskolin/IBMX + VX-770-mediated response was not modified in genotypes with absent basal CFTR function associating F508del-CFTR with PTC mutations, 711 + 1 G > T or N1303K (Fig. 4b). In contrast, the F508del/1717-1 G > A and F508del/394delTT genotypes were strongly corrected, respectively by 11.2% and 29.6% of the average WT-CFTR level. Interestingly, the genotypes with strong initial residual Cl^−^ secretion, such as F508del/D1152H and L997F/R258G, displayed considerable correction, (respectively 40% and 74.6% from the basal value) reaching the WT value. This is significantly different from that observed in the F508del homozygote HNEs (18%).

Pooling the I_sc_ data from all the genotypes showed that the level of correction by lumacaftor was significantly correlated with the presence of residual basal CFTR-dependent Cl^−^ secretion (Fig. 5a). This was significantly correlated with the increase of CFTR expression at the apical face of the epithelium, as illustrated in Fig. 5b (and Supplementary Fig. S2). To investigate whether this could be related to culture dependent variation, we assessed the proportion of ciliated cells, the transepithelial resistance (R_T_) and the height of the epithelial layer. We did not find any difference for those parameters between the DMSO and the lumacaftor treated filters, nor between the “responder” (i.e. above 10% of the WT average Cl^−^ secretion) and the “non-responder” samples. This was further assessed by the absence of significant correlation between ΔI_scF/I+V_ and either R_T_ (*R* ^*2*^ = 0.0005, NS) or proportion of ciliated cells (*R* ^*2*^ = 0.38, NS). This suggests that the correction effect was not due to differences in cell differentiation.

**Figure 5:**
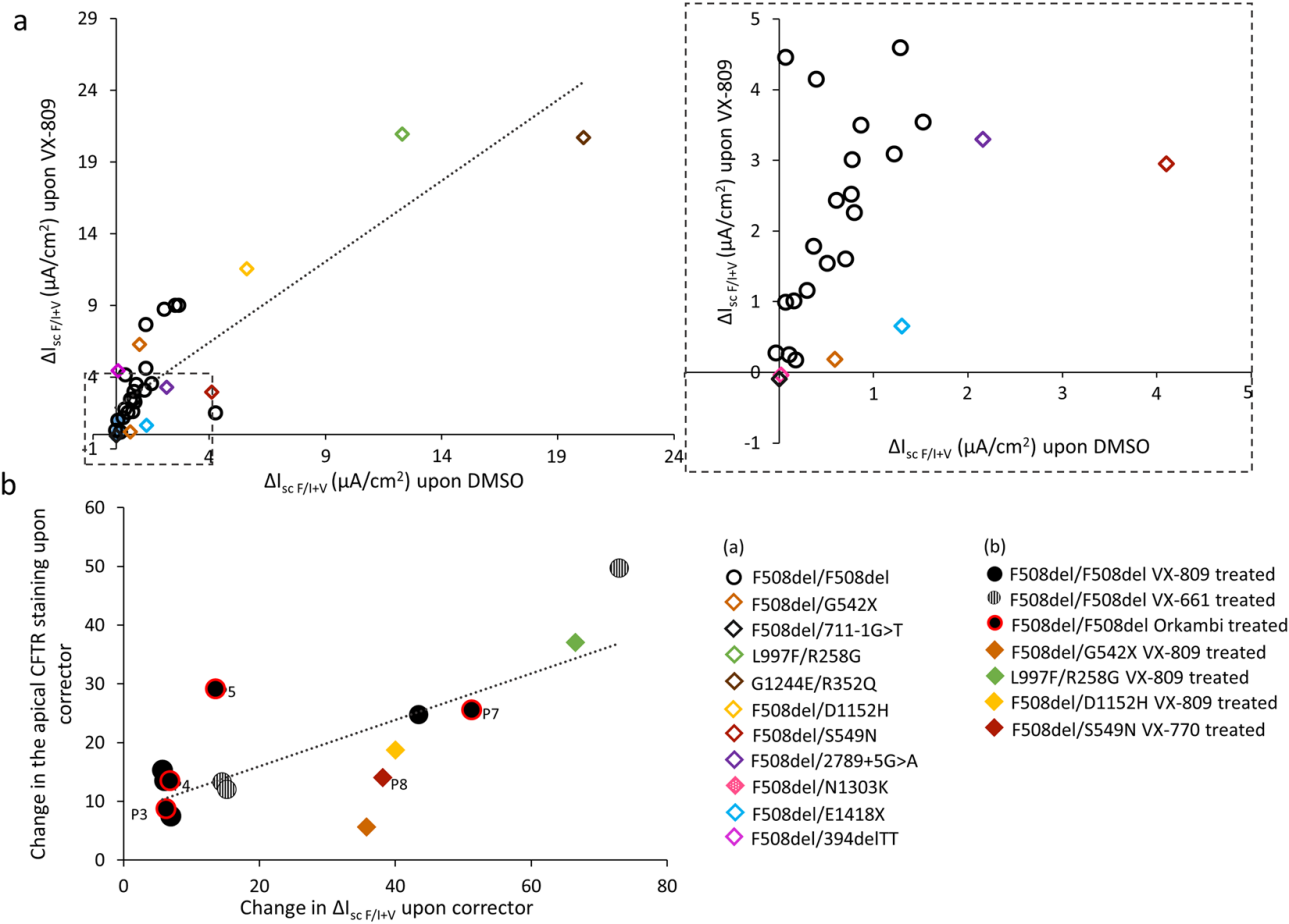
Lumacaftor stimulated CFTR activity correlates with basal CFTR activity among different genotypes. (**a**) Correlation of lumacaftor-stimulated CFTR activity (y-axis, ∆I_scF/I+V_ after treatment for 48 h at 37 °C with lumacaftor 3 µM) and basal CFTR activity (x-axis, ∆I_scF/I+V_ after treatment for 48 h with DMSO). The dotted line shows simple regression analysis of the data (*R* ^*2*^ = 0.74, *p* < 0.0001). Legend included in the figure. Inset showing the specific values for ∆I_scF/I+V_ < 5 µA/cm^2^. (**b**) CFTR corrector-stimulated CFTR activity changes (x-axis, ∆I_scF/I+V_) correlated with changes in the apical CFTR expression (y-axis, apical CFTR staining). The CFTR activity change is the difference between ∆I_scF/I+V_ after treatment for 48 h at 37 °C with lumacaftor 3 µM and ∆I_scF/I+V_ after treatment for 48 h with DMSO, expressed as percentage of the mean ∆I_scF/I+V_ value in wt/wt cells. Apical CFTR expression change is presented as the difference between the apical CFTR staining after treatment for 48 h at 37 °C with lumacaftor 3 µM and the apical CFTR staining after treatment for 48 h with DMSO, expressed as percentage of the mean apical CFTR staining in wt/wt cells. The dotted line shows simple regression analysis of the data (*R* ^*2*^ = 0.6, *p* < 0.001; n = 15). Legend included in the figure. Circular points indicate F508del/F508del cells (black filled circles: lumacaftor treated; circles with lines: tezacaftor treated; black filled circles with red frame: Orkambi treated; HNEs obtained from CF patients before starting the Orkambi treatment are indicated by the letter P and a number), filled rhomboid points: other CF genotypes treated with lumacaftor or ivacaftor for P8.

The average correction of ∆I_scF/I+V_ was not significantly different between lumacaftor and tezacaftor, both for F508del homozygous genotypes, however, on an individual basis, the effect of the two molecules may be different (NS, paired signed rank comparison) (Supplementary Fig. S3b).

We finally evaluated lumacaftor on healthy wt/F508del heterozygous HNE cells from 5 donors. The average increase of ΔI_scF/I+V_ upon lumacaftor treatment was 23% (SEM = 16) of the average WT-CFTR level. Moreover, like in CF F508del/N1303K cells, the HNE with wt/N1303K genotype did not respond to lumacaftor treatment (Fig. 4b).

### *In vitro* assessment of CFTR functional modulation can be predictive of the clinical response in patients

CFTR function correction in HNE cultures was compared to its clinical efficacy, as assessed by the drug induced change of FEV_1_ at 6 months in 7 F508del homozygous patients treated with Orkambi and F508del/S549N patient treated with ivacaftor (Kalydeco) (Fig. 6).

**Figure 6:**
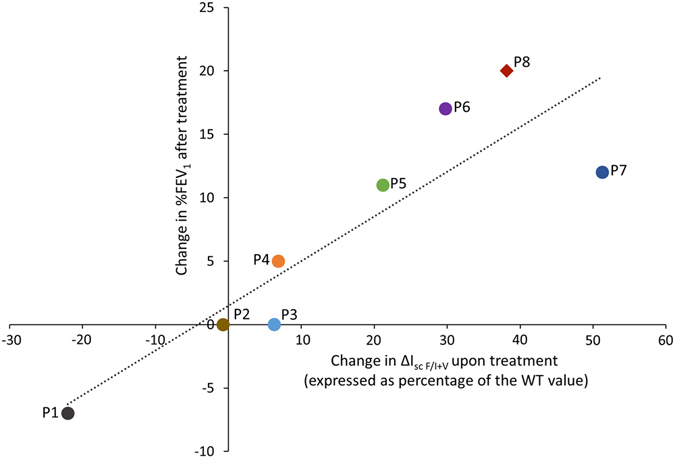
Correlation of CFTR modulators stimulated CFTR activity with changes in FEV_1_. Difference between ∆I_scF/I+V_ upon lumacaftor (3 µM) + ivacaftor (100 nM) or ivacaftor (100 nM) alone (for Patient 8) for 48 h at 37 °C and ∆I_scF/I+V_ upon DMSO, expressed as percentage of the mean ∆I_scF/I+V_ value in wt/wt cells. Data from 7 F508del/F508del patients (P1 to P7) treated with Orkambi and patient (P8) carrying the F508del/S549N genotype treated with VX-770 ivacaftor. Correlation with changes in % FEV_1_ after 6 months of treatment with Orkambi (P1 to P7) or ivacaftor (P8), respectively. The value of change in %FEV_1_ upon treatment was obtained by the ratio of the absolute FEV1 value at 6 months of treatment minus FEV1 value just before starting treatment over absolute FEV1 value just before starting treatment for each individual patient. The value of change in ΔI_scF/I+V_ upon treatment was obtained by the following formula: ((ΔI_scF/I+V_ CFTR modulator – ΔI_scF/I+V_ DMSO)/mean ΔI_scF/I+V_ WT-CFTR) * 100. The two obtained values for each individual patient were used to calculate the correlation and generate the graphics with StatView software. The dotted line shows simple regression analysis of the data (*R* ^*2*^ = 0.95; *p* < 0.0001). Circular points: F508del homozygous patients treated with Orkambi; rhomboid point: F508del/S549N patient treated with ivacaftor. Different colors correspond to different patients.

Patients 1, 2 and 3 were considered non-responders due to decreased FEV_1_ by 7% or no change of FEV_1_, respectively, after 6 months of treatment (Fig. 6). Patient 4 showed moderate 5% improvement of FEV_1_ at 6 months of Orkambi treatment. Patients 5, 6 and 7 were considered responders because their respiratory function increased by more than 10% (Fig. 6).

CFTR activity correction upon Orkambi treatment in HNE cell cultures, as assessed by the drug induced change of the response to Forskolin/IBMX + VX-770, correlated significantly with the FEV_1_ change induced by drug (*R* ^*2*^ = 0.95, *p* < 0.0001) (Fig. 6 **)**. ∆I_scF/I+V_ did not vary in HNE cells from Patients 1, 2, and 3 who did not improve clinically. The highest improvement in Cl^−^ secretion was measured in Patients 6 and 7, with ∆I_scF/I+V_ values reaching 33% and 61%, respectively, of the average WT-CFTR function. This was correlated with the increase in apical CFTR expression (Fig. 5b). All patients who exhibited correction of their respiratory function above 5% displayed a mean change of ∆I_scF/I+V_, expressed as the percentage of the average WT level, above 10%.

The case report of Patient 8, carrying the F508del/S549N genotype (Fig. 6), illustrates these observations. The patient underwent a life-threatening exacerbation after hemoptysis, justifying lung transplant indication. Treatment with ivacaftor resulted in acute, impressive improvement in respiratory function, which avoided lung transplantation and greatly increased quality of life (Supplementary Fig. S4a,b). *In vitro* evaluation demonstrated no significant increase in the immunostaining of apical CFTR upon ivacaftor (Fig. 5b) but a strong effect of ivacaftor on I_scF/I+V_, reaching 60% (correction by 38%) of average WT-CFTR function (Supplementary Fig. S4c).

## Discussion

Our study demonstrates that primary HNE cell cultures recapitulate the properties of primary HBE cultures from both CF and non-CF individuals.

To obtain the target level of correction, we first focused on HNE cells from healthy controls and on F508del homozygous patients, as this is the most frequent mutation. We did not find any marked differences between HBE and HNE cells in terms of cell differentiation, electrical resistance, and CFTR function/expression, as already reported by McDougall *et al*.^19^ and Devalia *et al*.^25^. The increased Na^+^ transport of wt/wt HBE *versus* HNE cells, probably reflects the increase in the ENaC relative expression changes from the proximal to distal regions of the human respiratory tracts^26^. We also verified that there was no significant change in CFTR bioactivity after expansion of HNE at the first passage, as also recently reported by Gentzsch *et al*.^27^, and supported by transcriptomic data that did not evidence any changes in CFTR expression^28^.

Our study is the first to focus on the range of CFTR function and expression in F508del homozygous HNE cells. We demonstrated that the variability in the cAMP-dependent Cl^−^ transport was mainly due to inter-subject variability. We aimed to limit the intrinsic variability of experimentation from human sampling. To avoid this we standardized as much as possible the experiments by evaluating cells at the same passage 1, eliciting cultures with the same level of trans-epithelial resistance. This was also the reason why we decided to use first the non-reprogrammed cells. Indeed this strategy might have displayed an additional level of variability, linked to differences in the seeding composition of progenitors, as recently speculated^28^.

Resampling different subjects (1 healthy control, 1 F508del homozygous subject, and 1 F508del/E1418X subject) confirmed that the Forskolin/IBMX + VX-770 responses variability was limited. Moreover, values for R_T_, inhibition of Amiloride-sensitive Na^+^ currents, and activation of cAMP-dependent Cl^−^ currents were in the range of already published values for primary HNE cultures^17, 29–33^ sampled from healthy donors (R_T_ ranging from 427 ± 31 to 964 ± 545 Ωcm^2^ ^17, 29, 33^; ∆I_scAmiloride_ ranging from 23.9 ± 8.5 to 28 ± 3 µA/cm^2^ ^17, 33^; ∆I_scF/I+V_ ranging from 7 ± 2 to 16.1 ± 3.8 µA/cm^2^ ^17, 31, 33^) and F508del homozygous patients (R_T_ ranging from 542 ± 65 to 633 ± 256 Ωcm^2^ ^17, 33^; ∆I_scAmiloride_ ranging from 35 to 61 ± 8 µA/cm^2^ ^31, 33^; ∆I_scF/I+V_ ranging from 0.1 ± 0.1 to 0.7 ± 0.2 µA/cm^2^ ^17, 31, 33^. Furthermore, the response to Forskolin/IBMX + VX-770, display a relatively low variability both in healthy subjects (mean: 11.6 µA/cm^2^; SEM: 1.7) and F508del homozygotes (mean: 0.2 µA/cm^2^; SEM: 0.09). The fact that this response was able to differentiate genotypes with residual CFTR function supports the conclusion that the experimental variability is indeed mainly due to inter-individual and not inter-experimental variation.

The I_sc_ response to Forskolin/IBMX + VX-770, which allowed quantification of the CFTR-mediated Cl^−^ secretion measurements, was accurate enough to detect different levels of CFTR between controls, healthy heterozygotes, and patients with genotypes displaying from absent to residual CFTR function. We showed for the first time that the level of CFTR activity *in vitro* correlated with *in vivo* CFTR activity, as measured by NPD. Finally, and most importantly, this CFTR functional spectrum was related to a gradient in CFTR expression at the apical membrane. Altogether, these findings represent strong support for the physiological relevance of the evaluation of CFTR-mediated Cl^−^ transport in HNE cell cultures.

We assessed CFTR correction by lumacaftor and tezacaftor in a large set of F508del homozygous HNE cell cultures and evidenced a substantial variability. This observation cannot be related to the number of passages – all the HNE were assessed at passage 1- nor to the differentiation pattern as the transepithelial resistance, the height of the epithelial layer and the proportion of ciliated cells were similar among the “responder” and the “non-responder” samples. Interestingly, the level of correction correlated to the basal level of CFTR activity suggesting that other causes may be considered, such as associations of F508del with other mutations or CFTR polymorphisms which may affect CFTR folding or post-transcriptional modification modulating CFTR biogenesis^34^.

Interestingly, compound F508del heterozygotes genotypes displayed a significant correction after lumacaftor or tezacaftor treatment. This was unexpectedly the case for deletion and severe splicing mutations^35^, but also observed for mutation associated with atypical CF (D1152H)^36^. This broadens the population of patients that could benefit from lumacaftor or tezacaftor.

Most importantly, our study is the first to provide evidence that quantification of the CFTR-mediated Cl^−^ secretion in patient-derived HNE cells may be a valuable surrogate biomarker to predict the clinical response. Indeed, we found that only patients whose HNE cells displayed an increase in ∆I_scF/I+V_ above 10% of the WT level, exhibited significant clinical improvement at 6 months. The threshold of 10% was considered clinically relevant, as it is associated with a mild disease correlated to higher rates of pancreatic sufficiency, less elevated sweat chloride levels and milder respiratory manifestations^22, 24^. Conversely, the patients whose cells did not display any significant correction did not improve clinically.

Such data are particularly relevant in the context of emergent therapies that rescue CFTR to identify subjects with CF who may benefit from CFTR-modulating drugs. This has been already shown in organoid cultures derived from the rectal epithelia. Indeed, Dekkers *et al*., showed that the Forskolin induced swelling response correlated to some extent to the expected level of CFTR activity^37^. CFTR function and responses to ivacaftor and lumacaftor was studied in subjects with CF, who expressed a broad range of *CFTR* mutations. *In vitro* drug responses in rectal organoids positively correlated with published outcome data from clinical trials with lumacaftor and ivacaftor. Importantly, selecting two subjects expressing an uncharacterized rare *CFTR* genotype the authors showed a correlation between the clinical responses to treatment with ivacaftor and *in vitro* measurements of CFTR function in patient-derived rectal organoids. However, it must be pointed out that the proteomic and transcriptomic background in intestine is different from that of the respiratory epithelium. As the target tissue of the therapy is the lung, it is mandatory to derive also a surrogate marker for clinical efficacy from the respiratory epithelium.

Indeed, implementation of a preclinical biomarker, such as the quantification of CFTR-mediated Cl^−^ secretion in patient-derived HNE cells might allow the right drug to be targeted for the right patient. This will also allow detection of patients with other genotypes than F508del that are responsive to corrector therapies. This new classification, based on “theratype” and not only genotype^34^, is driven by patient-specific responses, thereby providing a rationale for a personalized medicine strategy tailored for every CF patient.

## Conclusion

Our study provides evidence that the functional restoration of CFTR in HNE cell cultures is appropriate to predict the clinical benefit from a CFTR modulator. This individualized medicine strategy would allow “good” responders for a specific drug to be targeted and treated, thereby providing the drug only to patients predicted to benefit from and repurpose the non-responders to clinical trials with other compounds. This constitutes the basis for a future personalized therapy strategy for CF, which will have a huge influence in bringing medicines to all patients. This heralds a new era in CF care where therapy will address the basic defect of the disease.

## Material and Methods

### Subjects

CF patients, healthy heterozygous subjects, and healthy non-CF carriers were recruited for HNE sampling (Clinical Trials: NCT02965326). The exclusion criteria were smoking, local nasal treatment and rhinitis at the time of sampling. The study was approved by the Ile de France 2 Ethics Committee, and written, informed consent was obtained from each adult and parent. (AFSSAPS (ANSM) B1005423-40, n° Eudract 2010-A00392-37; CPP IDF2: 2010-05-03-3). All experiments were performed in accordance with the guidelines and regulations described by the Declaration of Helsinki and the low Huriet-Serusclat on human research ethics. HBE cells were derived from lung explants after written informed consent from CF and non-CF subjects. In a second part of study, patients treated with Orkambi or Kalydeco were evaluated for respiratory improvement after at least 6 months of treatment. In this subgroup, nasal cells were always sampled before starting the modulator treatment.

### Primary bronchial and nasal epithelial cell sampling and culture

HNE cells were sampled by nasal brushing of both nostrils after local visualization of the nasal mucosa. HBE cells were isolated from bronchial explants as previously described^38^. Freshly isolated HBE or HNE cells were then seeded on porous filters (0.33 cm^2^, Transwell, Corning) and supplemented with culture medium. After 2 days, cells were cultured in an air-liquid interface for 3–4 weeks. At least 2 filters were analyzed per patient to assess reliable results.

To increase the number of filters available to test the corrector efficacy, we used conditionally reprogrammed and re-differentiated HNE cells^39^. CFTR modulators, VX-809 lumacaftor (3 µM) and VX-661 tezacaftor (3 µM) dissolved in DMSO, were added to the basal side of polarized HNE or HBE cells grown at an air-liquid interface and incubated during 2 days in 5% CO_2_ at 37 °C, before experimentation. To evaluate the HNE cells of patients treated with Orkambi we also tested the association of lumacaftor with ivacaftor (100 nM) for 48 h. Patient’s cells treated with DMSO constituted their own control. The supplementary information provides further details about this method.

### Ussing chamber studies

The short-circuit-current (I_sc_) was measured under voltage clamp conditions with an EVC4000 Precision V/I Clamp (World Precision Instruments). For all measurements chloride concentration gradient across the epithelium was applied by differential composition of basal and apical Ringer solutions. The basal Ringer solution contained: 145 mM NaCl, 3.3 mM K_2_HPO_4_, 10 mM HEPES, 10 mM D-Glucose, 1.2 mM MgCl_2_, and 1.2 mM CaCl_2_ and apical solution contained: 145 mM Na-Gluconate, 3.3 mM K_2_HPO_4_, 10 mM HEPES, 10 mM D-Glucose, 1.2 mM MgCl_2_, 1.2 mM CaCl_2_. Inhibitors and activators were added after stabilization of baseline I_sc:_sodium (Na^+^)-channel blocker Amiloride (100 µM) to inhibit apical epithelial Na^+^ channel (ENaC); cAMP agonists Forskolin (10 µM) and 3-isobutyl-1-methylxanthine (IBMX 100 µM) to activate the transepithelial cAMP-dependent current (including Cl^−^ transport through CFTR channels); VX-770 (10 µM) to potentiate CFTR activity; CFTR inhibitor CFTR_inh_172 (5 µM) to specifically inhibit CFTR; and ATP (100 µM) to challenge the purinergic calcium-dependent Cl^−^ secretion. The sum of the change after Forskolin/IBMX and VX-770 (∆I_scF/I+V_) served as an index of CFTR function. The supplementary information provides further details about this method.

### Immunocytochemistry

CFTR immuno-detection was performed as previously described^40^. Apical CFTR staining was assessed semi quantitatively as the percentage of cells displaying apical staining multiplied by the average corrected apical fluorescence^41^. The supplementary information provides further details about these methods.

### Nasal potential difference (NPD) measurement

NPD measurement was performed as described previously^42^. The sum of ∆low chloride and ∆Isoproterenol (∆LowCl^–^Iso) served as an index of CFTR function. The supplementary information provides further details about this method.

### Statistical analysis

Statistical analysis was performed using S.A.S software. As several HNE/HBE filters were obtained from a single patient, quantitative parameters were expressed as median values per patient. Means (±SEM) were calculated from the median values per patient. Comparisons between groups were carried out using the Mann-Whitney U test. Correlation coefficients were calculated by Spearman’s rank correlation. *R* ^*2*^ coefficients were calculated to assess how the data fitted to the regression model.

As the number of cultures tested for each patient varied, a linear random-effects model was used to decompose inter- and intra-patient variability. The part of inter-patient variability was expressed by the intra-class correlation coefficient defined as the ratio of inter over inter + intra variability estimates. Lumacaftor efficacy in the F508del/F508del patients was evaluated with the mixed effect linear model, using treatment as the fixed effect and the patient as the random effect to consider the inter-patient and inter-filter variability. All statistical tests were two-sided and a *p*-value < 0.05 was considered statistically significant.

## Electronic supplementary material

Supplementary Information
