# Supplementary material for: Correction of CFTR function in nasal epithelial cells from cystic fibrosis patients predicts improvement of respiratory function by CFTR modulators
Source: Sci Rep. 2017 Aug 7;7:7375. doi: 10.1038/s41598-017-07504-1 (PMC5547155; doi:10.1038/s41598-017-07504-1)

Correction of CFTR function in nasal epithelial cells from cystic fibrosis patients predicts improvement of respiratory function by CFTR modulators

Iwona M Pranke[1], Aurélie Hatton[1], Juliette Simonin[1], Jean Philippe Jais[2], Françoise Le Pimpec-Barthes[3], Ania Carsin[4], Pierre Bonnette[5], Michael Fayon[6], Nathalie Stremler-Le Bel[4], Dominique Grenet[5], Matthieu Thumerel[7], Julie Mazenq[4], Valerie Urbach[1], Myriam Mesbahi[1], Emanuelle Girodon-Boulandet[8], Alexandre Hinzpeter[1], Aleksander Edelman[1] and Isabelle Sermet-Gaudelus [1,9,*]

Supplementary Material and Methods

Primary bronchial and nasal epithelial cells sampling and culture

HNE cells were sampled by nasal brushing of the medial wall and the inferior turbinate of both nostrils and placed in DMEM/F-12 (Dulbecco's Modified Eagle's /Ham's F-12 media 50/50). Cells were then detached from the brush and centrifuged (1500g, 7 min, 4°C). After trypsinization, cells were re-suspended in an appropriate volume of amplification medium (DMEM/F-12, 5% fetal bovine serum, non-essential amino acids 1%v/v, Piperacillin/Tazobactam 90 µg/10 µg/ml, Amphotericin B 5 µg/ml) and counted.

HBE cells were isolated from bronchial explants (1-2 bifurcation) by enzymatic digestion, as previously described[1]. First, bronchial explants were washed twice with washing medium (Minimum Essential Medium Eagle (MEM) + Piperacillin/Tazobactam 90 µg/10 µg/ml, Ciprofloxacin 20 µg/ml, Amphotericin B 5 µg/ml + DTT 0.5 mg/ml + DNAse 10 µg/ml) followed by at least two washings with MEM containing only antibiotics (Piperacillin/Tazobactam 90 µg/10 µg/ml, Ciprofloxacin 20 µg/ml and Amphotericin B 5 µg/ml). Cells were then incubated for 24 h in medium MEM containing Piperacillin/Tazobactam 90 µg/10 µg/ml, Ciprofloxacin 20 µg/ml, Amphotericin B 5 µg/ml and protease 1 mg/ml. Finally, after neutralization of protease with serum, epithelial cells were scraped with scalpel and handled as HNE cells.

For air-liquid interface (ALI) culture of non-amplified cells, about 330,000 HBE or HNE cells suspended in amplification medium were seeded on type IV collagen-coated porous filter with a 0.33-cm^2 surface (Transwell, Corning). UG (Ultroser G) 2% medium (DMEM/F-12, supplemented with 2% Ultroser G) containing Piperacillin/Tazobactam 90 µg/10 µg/ml and Amphotericin B 5 µg/ml was added to the basal side of filters. After two days, apical medium was aspirated and cells were cultured in ALI with UG 2% basal medium changed daily for 3-4 weeks to establish a differentiated epithelium[2,3].

The protocol for cell expansion was adapted from Suprynowicz et al.[4]. Conditional reprogramming was performed by co-culturing the cells with irradiated fibroblasts and Rho-Kinase inhibition and to minimize phenotypic changes, cells were differentiated after passage 1. Briefly, freshly isolated HNE cells were first grown on a feeder layer, co-cultured with irradiated NIH-3T3 fibroblasts in DMEM/F-12 cell culture medium supplemented with 10% newborn calf serum, Rho kinase inhibitor Y-27632 (10 µM), Piperacillin/Tazobactam 90 µg/10 µg/ml and Amphotericin B 5 µg/m. Cells were then supplemented with fresh culture medium every other day and after achieving ~2-3 $x10^6$ cell expansion, cultures were trypsinized to separate feeders and epithelial expanded cells.

The transepithelial electrical resistance (R_T) of cultures was measured with a chopstick voltmeter (Millicell-ERS) and only cultures with at least 600 Ω/cm^2 were considered for the following experiments.

CFTR modulators VX-809 (3 µM), VX-661 (3 µM) and VX-770 (100 nM) dissolved in DMSO were diluted in UG 2% medium and applied for 2 days at the basal side before experimentation.

Ussing chamber studies

Short circuit current (I_{sc}) was measured under a voltage clamp with an EVC4000 Precision V/I Clamp (World Precision Instruments). Culture inserts with differentiated HNE or HBE cells, were mounted in Ussing chambers (Physiologic Instruments, San Diego, CA). For all measurements chloride concentration gradient across the epithelium was applied by differential composition of basal and apical Ringer solutions. The basal Ringer solution contained: 145 mM NaCl, 3.3 mM K_2HPO_4, 10 mM HEPES, 10 mM D-Glucose, 1.2 mM $MgCl_2$, and 1.2 mM $CaCl_2$ and apical solution contained: 145 mM Na-Gluconate, 3.3 mM K_2HPO_4, 10 mM HEPES, 10 mM D-Glucose, 1.2 mM $MgCl_2$, 1.2 mM $CaCl_2$. During continuous recording of I_{sc} (in voltage-clamp mode), the following inhibitors and activators were added at the apical side after stabilization of baseline I_{sc}: sodium (Na^+)-channel blocker Amiloride (100 µM) to inhibit apical

epithelial Na^+ channel (ENaC); cAMP agonists Forskolin (10 µM) and 3-isobutyl-1-methylxanthine (IBMX 100 µM) to activate the transepithelial cAMP-dependent current (including Cl^- transport through CFTR channels); VX-770 (10 µM) to potentiate CFTR activity; CFTR inhibitor $CFTR_{inh}172$ (5 µM) to specifically inhibit CFTR; and ATP (100 µM) to challenge the purinergic calcium-dependent Cl^- secretion. The following parameters were then calculated: $\Delta I_{sc\ Amiloride}$ as the difference between I_{sc} after Amiloride and baseline I_{sc}; $\Delta I_{sc\ F/I}$ as the difference between I_{sc} after Forskolin/IBMX and I_{sc} after Amiloride; $\Delta I_{sc\ VX\text{-}770}$ as the difference between I_{sc} after VX-770 and I_{sc} after Forskolin/IBMX; $\Delta Isc_{\ CFTRinh172}$ as the difference between I_{sc} after $CFTR_{inh}172$ and I_{sc} after VX-770.

The sum of the change after Forskolin/IBMX and VX-770 ($\Delta I_{sc\ F/I+V}$) served as an index of CFTR function.

Immunocytochemistry

Polarized HBE and HNE epithelia were stained with wheat germ agglutinin coupled to Alexa Fluor 594 (WGA-Alexa Fluor 594) diluted in Hank's buffered salt solution (HBSS) buffer for 10 min at 37°C. Cells were then washed with HBSS and fixed with ice-cold acetone for 5 min, then rinsed twice with phosphate-buffered saline (PBS). Cells were then incubated in blocking solution (3% bovine serum albumin in PBS containing 0.1% Triton-X100) for 20 min. CFTR immuno-detection was performed with p.24-1 (R&D Systems) antibody (Ab) that recognizes the C-terminus of CFTR, diluted 1/100 in blocking solution during overnight incubation at 4°C and Mr Pink Ab recognizing the NBD1 domain (from CFTR Folding Consortium), diluted 1/50. Preliminary study showed that the latter antibody displayed similar pattern as p.24-1, but the staining was faint. We therefore decided to only use p.24-1 for further evaluation. CFTR immunostaining did not differ according to WGA pretreatment, which demonstrates that p.24-1 does not associate to WGA. Zona occludens–1 (ZO-1) (Santa Cruz Biotech, sc-10804, 1/500 dilution), alpha-tubulin (Abcam, ab80779, 1/300 dilution), Muc 5AC (Santa Cruz Biotech, sc-

20118, 1/250 dilution) and cytokeratin 8 (Progen, 61038, 1/250 dilution) staining were done on additional filters. After washing with PBS-Triton-X100 0.1%, goat secondary Ab conjugated to Alexa 488 (Invitrogen, A11001) or 594 (Invitrogen, A11012) was added for 30 min at 1/1000 dilution in 10% goat serum. After a final wash, Vectashield mounting medium containing DAPI (Vector Laboratories, H-1200) was used to mount cells on microscope slides.

A Leica TCS SP5 AOBS confocal microscope (63x/1.4 oil differential interference contrast λ blue PL APO objective) was used to capture images, which were analyzed with the ImageJ software (NIH). The 3D reconstitution of polarized epithelia of HBE/HNE cells was performed with the 3D Viewer plugin in Image J.

Apical CFTR staining was assessed semi quantitatively as the percentage of cells displaying apical staining multiplied by the average corrected apical fluorescence. For this parameter, readings were taken in the apical zone of at least 3 epithelial cells and in the region outside the cells for background estimation. The average corrected apical fluorescence was then calculated, using Image J software as **"**Integrated density – (Area of selected cell x Mean fluorescence of background readings)**"** [5]**.**

Nasal potential difference measurements

Nasal potential difference (NPD) measurements were performed as described previously[6], according to sequential perfusion of 100 µM Amiloride in saline solution (ΔAmiloride); Amiloride in low-chloride solution to drive chloride secretion (Δlow chloride); Amiloride plus 10 µM Isoproterenol in low-chloride solution to stimulate the cAMP-dependent Cl^- conductance related to CFTR (ΔIsoproterenol). The sum of Δlow chloride and ΔIsoproterenol (ΔLowCl^--Isoproterenol) served as an index of CFTR function.

Supplementary Results

Correlation between fluorescence intensity and percentage of apical CFTR staining

Supplementary Figure S2 shows the correlation between the average corrected apical fluorescence intensity and the percentage of cells displaying apical staining according to genotypes in the whole population (R^2=0.7, p<0.0001; simple regression analysis).

Detailed response to Forskolin/IBMX + VX-770 in HNE cells in non F508del/F508del CF genotypes

$\Delta I_{scF/I+V}$ was barely detectable, below 5% of the WT-CFTR response, for the cultures with genotypes homozygous for a premature termination codon (PTC) (Y122X and G542X) or compound heterozygote F508del in *trans* with a nonsense mutation (G542X), a deletion generating a PTC (394delTT), a mutation associated with abnormal protein folding (N1303K), or splicing mutations resulting in little protein production (711+1G>T, 1717-1G>A). In contrast, the F508del/E1418X genotype, with a PTC in the penultimate exon[7], displayed a residual CFTR function at 10.3% of the WT-CFTR level. Similarly, when F508del was associated with 2789+5G>A, a mild class V mutation associated with residual normal exon 16 splicing[8], this provided a residual Cl^- transport at 14.6% of the WT-CFTR value. Finally, the cells compound heterozygotes for F508del and D1152H, a class IV regulation defect associated with atypical CF[9], displayed a strong residual Cl^- transport after Forskolin/IBMX+VX-770 stimulation. This was also the case for the F508del-R117H-T7 genotype, which has been demonstrated to retain a Cl^- conductance[10], where a normal $\Delta I_{scF/I+V}$ change was evidenced. Two genotypes involving mutations of uncertain liability L997F/R258G[11] and G1244E/R352Q[12] displayed a WT-CFTR level.

VX-809 did not modify the $\Delta I_{scF/I+V}$ in genotypes with absent basal CFTR function associating F508del CFTR with PTC mutations, 711+1G>T or N1303K. In contrast, the F508del/1717-

1G>A genotype was strongly corrected, reaching 15.4% of the WT-CFTR level. Similarly, the CFTR activity of the F508del/394delTT HBE cells was increased to 30% of WT-CFTR activity. Interestingly, the genotypes with strong initial residual Cl^- secretion, such as F508del/D1152H and L997F/R258G, displayed considerable correction, allowing the WT-CFTR value to be reached. The only exception was the F508del/2789+5G>A, which displayed only a 7.7% increase of $\Delta I_{scF/I+V}$ upon VX-809, however, this reached 22% of the average WT-CFTR level because of the basal residual function.

Supplementary Figures legend

Supplementary Figure S1. Differentiation and polarization of the air-liquid interface HNE cultures

Representative confocal microscopy images of immuno-fluorescent staining of cell markers performed after ice-cold acetone fixation. (a) Projections of confocal microscopy images, top views of Z stacks. Immunofluorescence staining of: Zona occludens-1 (ZO-1, tight junctions protein) in red, Cytokeratin 8 (K8, marker of epithelial cells) in green, Mucin 5AC (Muc5AC, marker of goblet secretory cells) in red, alpha-Tubulin in green, and CFTR in green (antibodies p.24-1 and Mr Pink as indicated) with accompanying DAPI staining of nuclei in blue. Scale bar = 20µm. (b) 3D reconstitutions from Z stacks. Immuno-fluorescent staining of ZO-1 in red, K8 in green, and DAPI (left image), α-Tubulin in green and DAPI in blue (right image). Scale bar in panel (a) applies to the images in panel (b). (c) Representative tracings of the short-circuit current (I_{sc}, µA/cm^2) response to 100 µM Amiloride, 10 µM Forskolin and 100 µM 3-isobutyl-1-methylxanthine (IBMX), 10 µM VX-770, and 5 µM CFTR$_{inh}$172 in reconstituted epithelia after 3 weeks of ALI culture (left panel: wt/wt cells; right panel: F508del/F508del cells).

Supplementary Figure S2. Correlation between the percentage of cells with apical CFTR staining and the average corrected apical fluorescence intensity according to genotype

Intensity of fluorescence was evaluated in the apical zone of at least 3 epithelial cells from 3 random fields and in the region outside the cells for background estimation. Data from 5 wt/wt control subjects, 11 F508del/F508del patients (DMSO and VX-809/VX-661 treatment for 2 days at 37°C), 3 wt/F508del (+ 1 wt/N1303K) heterozygotes and 11 patients with other genotypes (DMSO and VX-809 treatment for 2 days at 37°C). VX-809 treatment is indicated by filled symbols, VX-661 is indicated by dotted symbols.

The dotted line shows simple regression analysis of the data (R^2 for the whole dataset=0.7, p<0.0001).

Supplementary Figure S3. Tezacaftor-stimulated correction of CFTR-dependent Cl^- secretion

(a) Values of $\Delta I_{scF/I+V}$ after treatment for 48 h at 37°C with DMSO and VX-661 3 µM of HNE (solid line) and HBE (dashed line) cultures from different F508del/F508del patients (black) and patients with other CF genotypes (colors, indicated in the figure). Supplemental horizontal dashed lines in the graphs indicate the level of mean $\Delta I_{scF/I+V}$ in wt/F508del cultures sampled from healthy carriers with normal phenotype and 10% of the mean $\Delta I_{scF/I+V}$ value in wt/wt cultures corresponding to mild CF disease. (b) Comparison of VX-809 and VX-661 correction in HNE and HBE cells from 12 F508del homozygotes and 4 patients with other CF genotypes (indicated in figure). Values of $\Delta I_{scF/I+V}$ achieved after VX-809 (3 µM) or VX-661 (3 µM) treatment for 48 h at 37°C, expressed as percentage of the mean WT-CFTR level. Filled rectangles: VX-809; dotted rectangles: VX-661.

Supplementary Figure S4. Effect of ivacaftor and combination therapy on the F508del/S549N patient

(a) Representative computed tomography (CT) scan images taken before and after 1 and 6 months of treatment with VX-770 (ivacaftor) are shown; (b) Forced expiratory volume in 1 s at different time points. (c) $\Delta I_{scF/I+V}$ measured in HBE cells obtained from F508del/S549N patient, after 48-h treatment at 37°C with various CFTR modulators as indicated on the figure: potentiator VX-770 (100 nM) and correctors VX-809 (3 µM) and VX-661 (3 µM) alone and in combination with VX-770, with DMSO as control. Reference $\Delta I_{scF/I+V}$ level at 50% of mean WT value is indicated with horizontal dashed lines. The maximum effect was obtained with

VX-770 or VX-661 alone which both displayed an increase in CFTR activity up to 60% of the WT range.

Supplementary Figure S5. Short-circuit-current and nasal potential difference recordings from wt/wt and wt/F508del nasal epithelium *in vitro* and *in vivo*

Representative tracings from I_{sc} measurements (upper panels) and NPD measurements (lower panels) in wt/wt nasal epithelium (a) and (b), and in wt/F508del nasal epithelium (c).

Supplementary Figure S6. Short-circuit-current and nasal potential difference recordings from F508del/F508del and F508del/G542X nasal epithelium *in vitro* and *in vivo*

Representative tracings from I_{sc} measurements (upper panels) and NPD measurements (lower panels) in F508del/F508del nasal epithelium (a) and (b), and in F508del/G542X nasal epithelium (c).

Supplementary Figure S7. Short-circuit-current and nasal potential difference recordings in nasal epithelium *in vitro* and *in vivo* in complex heterozygous patients

Representative tracings from I_{sc} measurements (upper panels) and NPD measurements (lower panels) in G1244E/R352Q (a) and L997F/R258G (b) nasal epithelium. Representative tracings from I_{sc} measurements in F508del/S549N (c) and F508del/D1152H (d) *in vitro* epithelium.

Supplementary Table 1. Minimal number of patients and filters required to test the efficacy of a corrector in HNE cultures isolated from F508del/F508del patients.

The calculation of the number of filters and patients per condition necessary for a given Power was based on the variability of the Forskolin/IBMX+VX-770 response. It postulated that the filters had to be divided equally among sequences.

No. of patients	No. of filters control/treated	Power
5	1/1	0.19
	2/2	0.48
	3/3	0.64
	4/4	0.78
10	1/1	0.35
	2/2	0.78
	3/3	0.92
20	1/1	0.76
	2/2	0.98
	3/3	0.995
30	1/1	0.88
	2/2	1
	3/3	1

Supplementary References

1. Gruenert, D. C., Finkbeiner, W. E. & Widdicombe, J. H. Culture and transformation of human airway epithelial cells. *Am. J. Physiol. - Lung Cell. Mol. Physiol.* **268,** L347–L360 (1995).
2. de Jong, P. M. *et al.* Ciliogenesis in human bronchial epithelial cells cultured at the air-liquid interface. *Am. J. Respir. Cell Mol. Biol.* **10,** 271–277 (1994).
3. Prulière-Escabasse, V. *et al.* TGF-beta 1 downregulates CFTR expression and function in nasal polyps of non-CF patients. *Am. J. Physiol. Lung Cell. Mol. Physiol.* **288,** L77-83 (2005).
4. Suprynowicz, F. A. *et al.* Conditionally reprogrammed cells represent a stem-like state of adult epithelial cells. *Proc. Natl. Acad. Sci. U. S. A.* **109,** 20035–20040 (2012).
5. McCloy, R. A. *et al.* Partial inhibition of Cdk1 in G 2 phase overrides the SAC and decouples mitotic events. *Cell Cycle Georget. Tex* **13,** 1400–1412 (2014).
6. Sermet-Gaudelus, I. *et al.* Clinical phenotype and genotype of children with borderline sweat test and abnormal nasal epithelial chloride transport. *Am. J. Respir. Crit. Care Med.* **182,** 929–936 (2010).
7. Gaitch, N. *et al.* CFTR and/or pancreatitis susceptibility genes mutations as risk factors of pancreatitis in cystic fibrosis patients? *Pancreatol.* **16,** 515–522 (2016).
8. Highsmith, W. E. *et al.* Identification of a splice site mutation (2789 +5 G > A) associated with small amounts of normal CFTR mRNA and mild cystic fibrosis. *Hum. Mutat.* **9,** 332–338 (1997).
9. Burgel, P.-R. *et al.* Non-classic cystic fibrosis associated with D1152H CFTR mutation. *Clin. Genet.* **77,** 355–364 (2010).
10. Sheppard, D. N. *et al.* Mutations in CFTR associated with mild-disease-form Cl- channels with altered pore properties. *Nature* **362,** 160–164 (1993).

11. Lucarelli, M. *et al.* A new complex allele of the CFTR gene partially explains the variable phenotype of the L997F mutation. *Genet. Med.* **12,** 548–555 (2010).

12. Guinamard, R. & Akabas, M. H. Arg352 is a major determinant of charge selectivity in the cystic fibrosis transmembrane conductance regulator chloride channel. *Biochemistry* **38,** 5528–5537 (1999).

Supplementary Figure S1

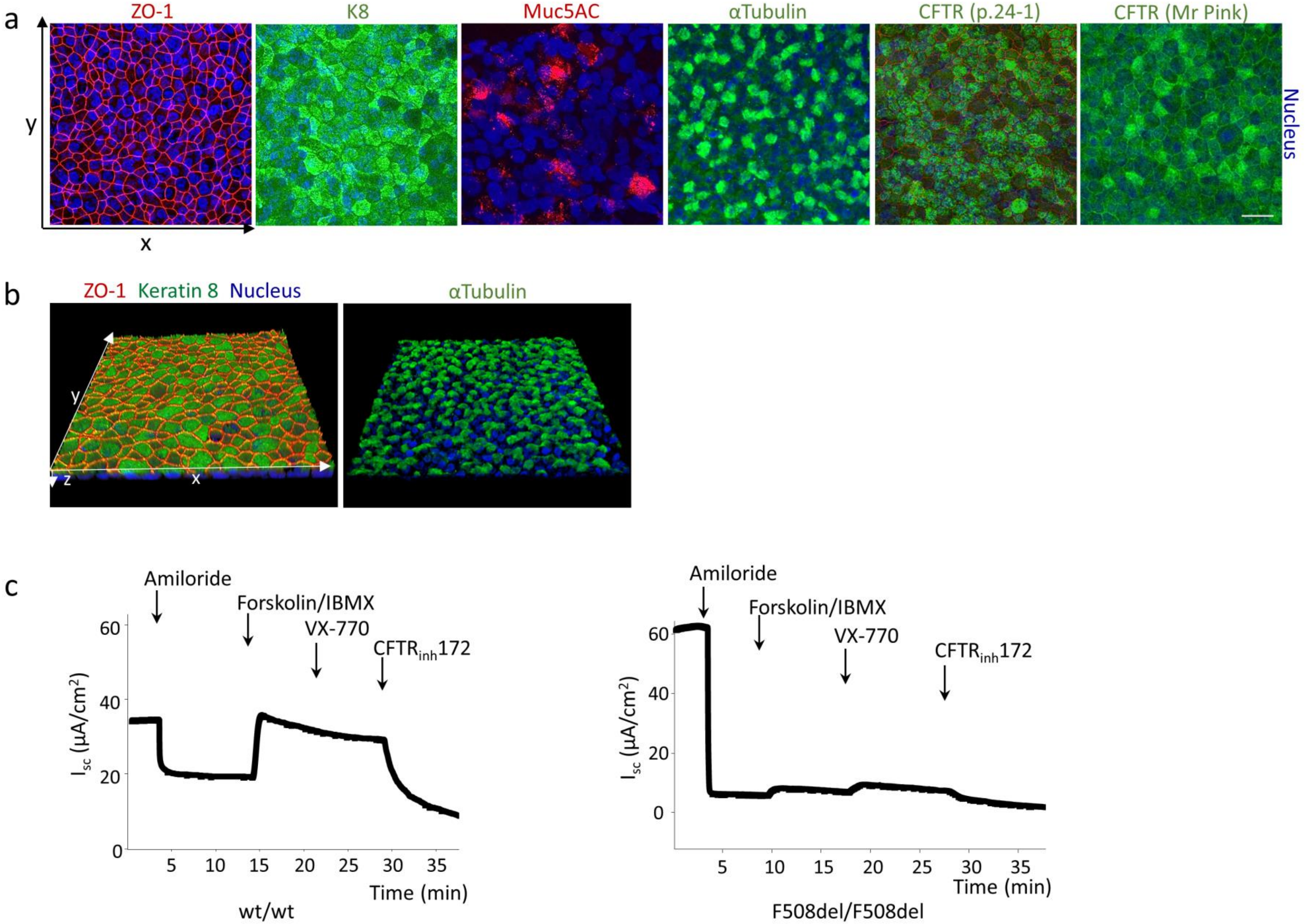

Supplementary Figure S2

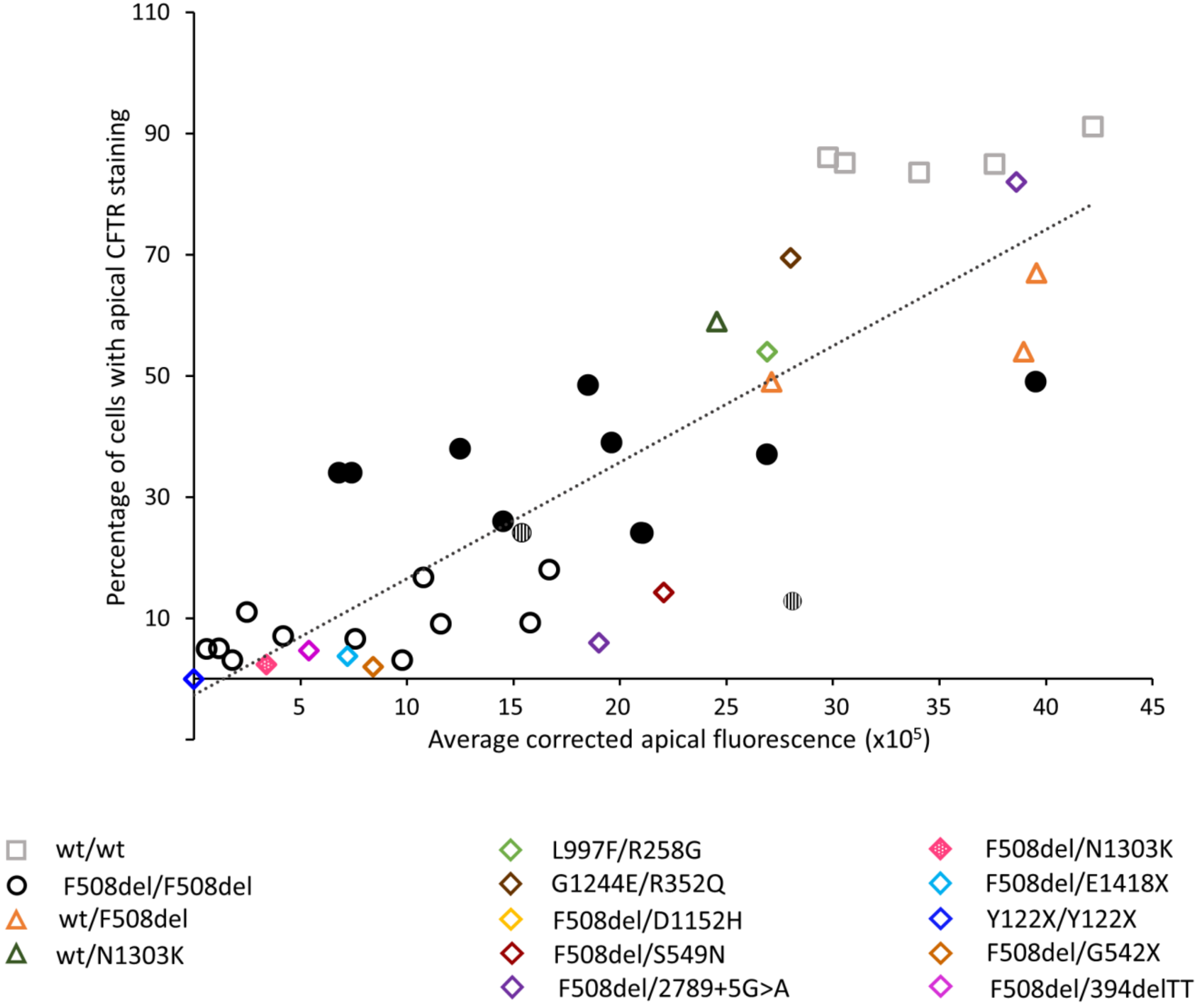

Supplementary Figure S3

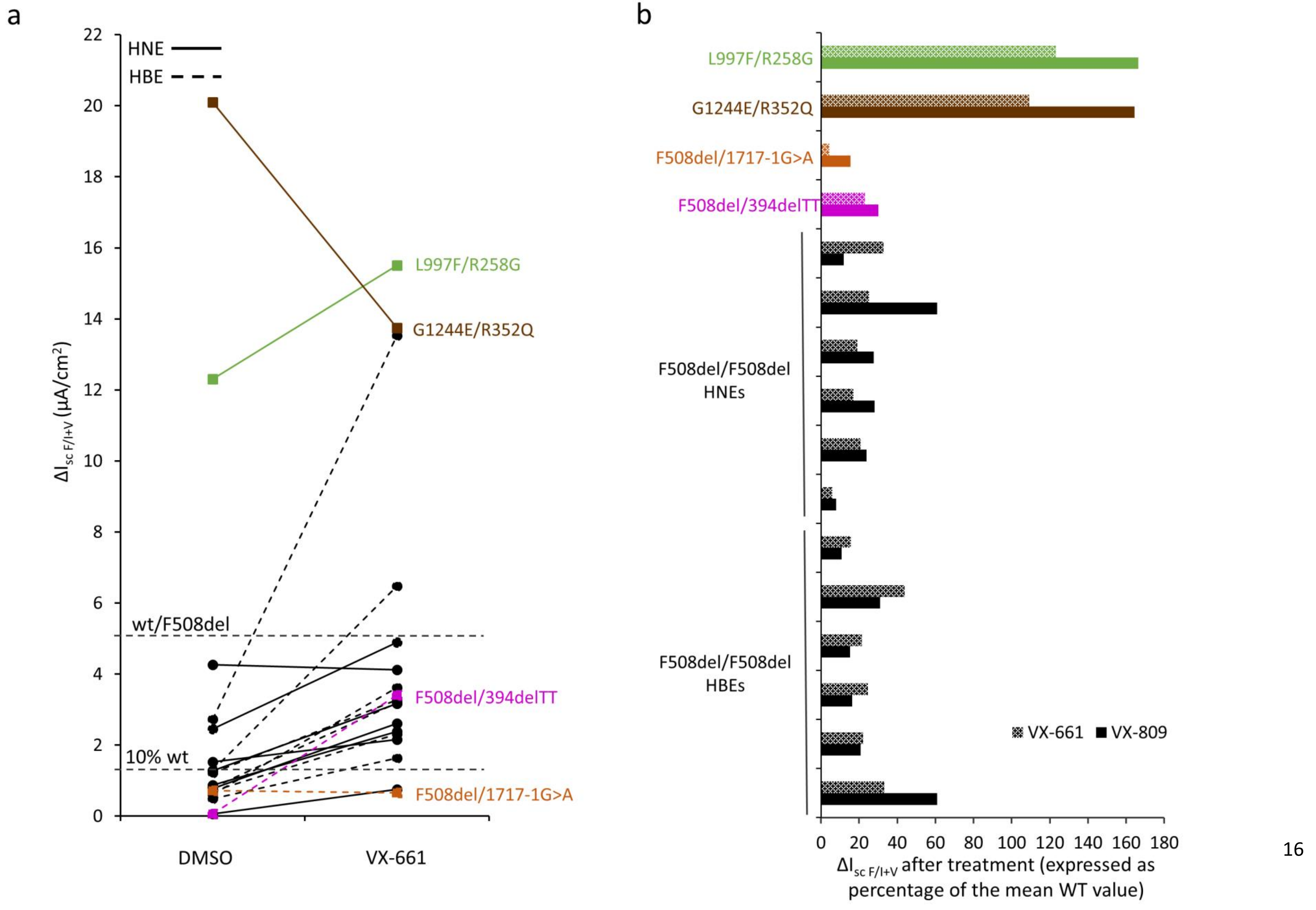

Supplementary Figure S4

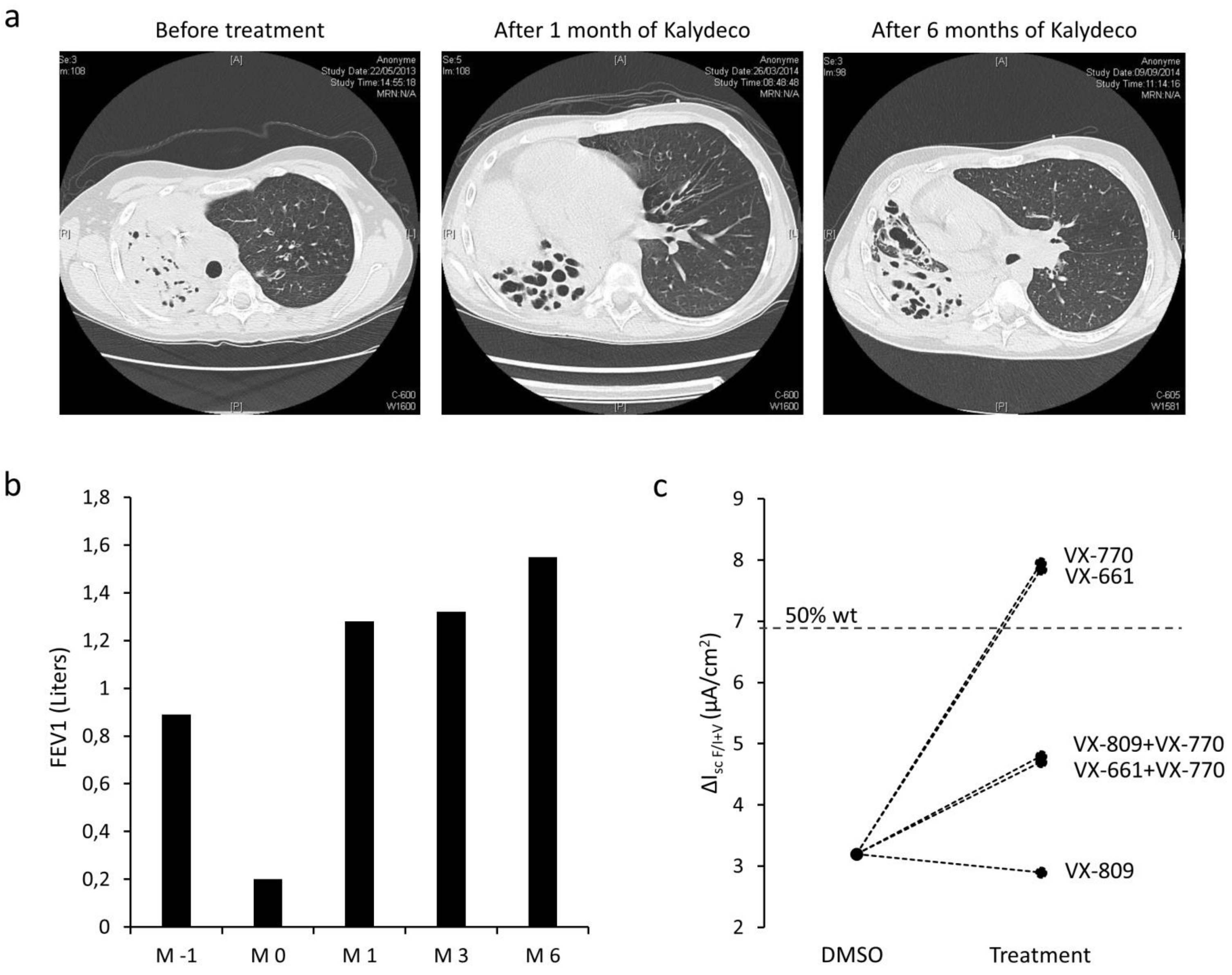

Supplementary Figure S5

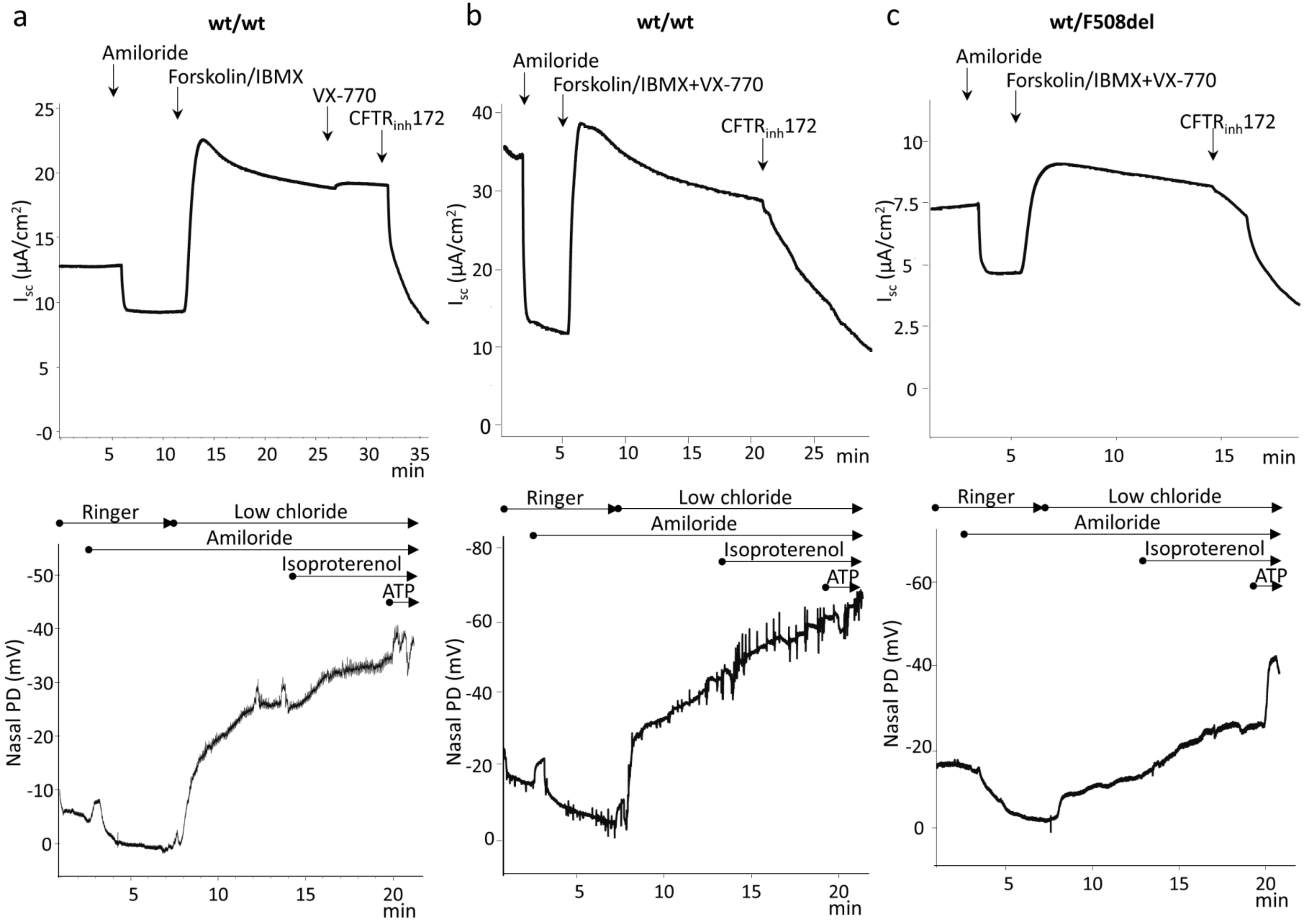

Supplementary Figure S6

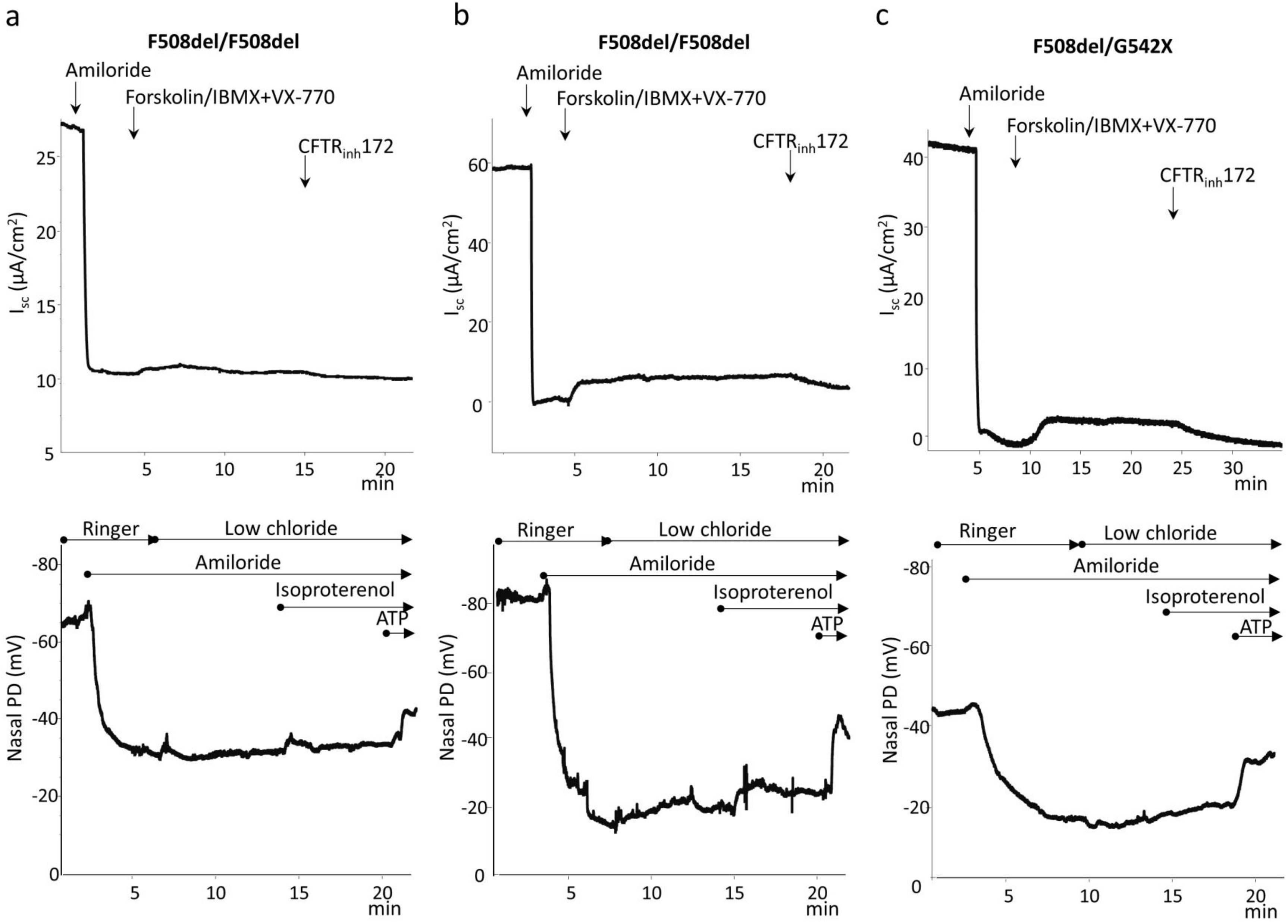

Supplementary Figure S7

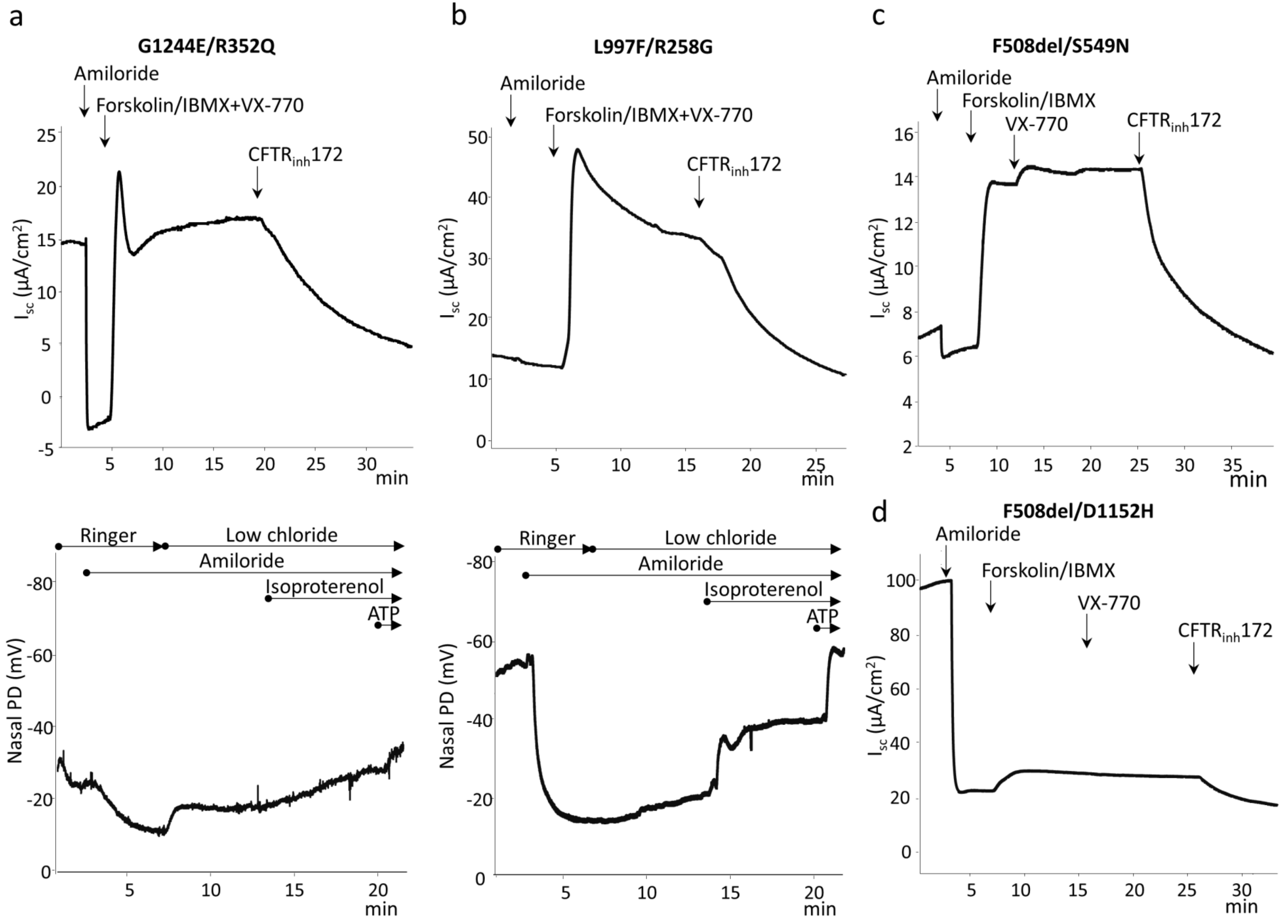

Supplement: Supplementary file 1 — Supplementary Information [file 41598_2017_7504_MOESM1_ESM.pdf]
